# A randomized controlled trial to verify the irrigation of salivary glands in relieving xerostomia in patients with Sjögren’s syndrome

**DOI:** 10.3389/fimmu.2022.1039599

**Published:** 2022-11-10

**Authors:** Hongming Du, Zhen Fu, Yi Zhong, Yi Yuan, Jing Zhao, Xu Ding, Sheng Li, Shiyu Gao, Yuchi Zhu, Haiyang Song, Heming Wu

**Affiliations:** ^1^ Department of Oral and Maxillofacial Surgery, Affiliated Stomatological Hospital of Nanjing Medical University, Nanjing, Jiangsu, China; ^2^ Jiangsu Key Laboratory of Oral Disease, Nanjing Medical University, Nanjing, Jiangsu, China; ^3^ Jiangsu Province Engineering Research Center of Stomatological Translational Medicine, Nanjing, Jiangsu, China; ^4^ Department of Stomatology, The Fourth Affiliated Hospital of Nanjing Medical University, Nanjing, Jiangsu, China; ^5^ Department of General Dentistry, Affiliated Stomatological Hospital of Nanjing Medical University, Nanjing, Jiangsu, China

**Keywords:** irrigation, saliva, salivary glands, Sjögren’s syndrome, xerostomia

## Abstract

**Objective:**

To verify the effect of triamcinolone acetonide (TA) and major salivary glands saline irrigation on relieving xerostomia in Sjögren’s syndrome (SS) patients.

**Methods:**

The enrolled 49 SS patients were randomly assigned to the control group (no irrigation, n=16), saline group (irrigation with saline, n=17) and TA group (irrigation with TA, n=16). Fourteen cases of each group were treated differently but received the same examinations. The examinations include unstimulated whole saliva flow (UWS), chewing-stimulated whole saliva flow (SWS), citric acid-stimulated parotid flow (SPF), Clinical Oral Dryness Score (CODS), Xerostomia Inventory (XI) and EULAR SS Patient Reported Index (ESSPRI) of 1 week before irrigation (T0) and 1 week(T1), 8 weeks (T8), 16 weeks (T16) and 24 weeks (T24) after major salivary irrigation.

**Results:**

Each group had 14 cases with completed follow-ups. Both TA and saline irrigation of major salivary glands resulted in higher SWS and SPF of T8, T16 and than those at T0. ESSPRI (oral dryness domain) of T8, T16 and T24 were significantly lower than that at T0, respectively (*P* < 0.05). SWS and SPF of T8, T16 and T24 in the saline group were significantly higher than in the control group (*P*< 0.05). XI and ESSPRI (oral dress domain) of T8, T16 and T24 in the saline group were significantly lower than those in the control group, respectively (*P*< 0.05). SWS and SPF of T16 and T24 in the TA group were significantly higher than in the control group (*P*< 0.05). All cases with completed follow-up in TA and saline groups were divided into responders and non-responders. Compared with responders, the UWS, SWS, SPF and CODS of T0 in non-responders were significantly increased (*P*<0.05). Compared with responders, the XI and ESSPRI of T0 in non-responders were significantly decreased (*P*<0.05).

**Conclusion:**

The irrigation of major salivary glands by TA and saline relieve xerostomia in SS patients. Patients with non-severe xerostomia (responders) have better relief after irrigation than patients with severe xerostomia (non-responders).

**Clinical Trial Registration:**

www.chictr.org.cn, identifier (ChiCTR210052314).

## 1 Introduction

Sjögren’s syndrome (SS)is a chronic inflammatory autoimmune disease involving exocrine glands (1, 2). Lymphocytes invade exocrine glandular epithelial cells of the lacrimal gland and salivary gland and lead to secretory dysfunction and subsequent clinical symptoms such as xerostomia and xerophthalmia (2, 3). The severe symptoms mentioned above can significantly affect the life quality of patients with SS (4–6).

At present, some clinical measures have been taken to alleviate xerostomia in SS patients. Artificial saliva and mucosal humectants are used to relieve xerostomia; M3 receptor agonists can also promote the secretion of saliva (7, 8). In addition, many patients treated with immunomodulators significantly relieve xerostomia when their systemic conditions are improved (9, 10). However, due to the short actuation duration, the above measures need to be used repeatedly. Systemic administration can also increase the incidence of side effects. Therefore, it is necessary to find an effective way that can promote saliva secretion in the long term with fewer side effects.

It has been reported that saline irrigation assisted by sialendoscopy can promote salivary secretion (11). The combined application of TA irrigation and sialendoscopy has achieved better therapeutic effects than irrigation with saline by sialendoscopy (12, 13).

Sialendoscopy can remove the stricture of the main duct to facilitate saliva outflow (14, 15). However, many salivary gland imaging studies have confirmed stenoses in each branch of the ductal system in patients with SS. In fact, morphological abnormalities in the main duct are rare, and the abnormalities are mainly dilations but not stenoses (16, 17). The application of sialendoscopy has also increased the incidence of complications (18). Therefore, this study will evaluate the effect of saline and TA irrigation on the function of salivary secretion in SS patients, respectively.

## 2 Methods

### 2.1 Patients enrollment

All patients included in this study were diagnosed as SS according to the 2002 American-European Consensus Group classification criteria (19). Participants were recruited from the Department of Oral and Maxillofacial Surgery, Affiliated Stomatological Hospital of Nanjing Medical University. The patients were aged between 18 to 75 years old, with unstimulated whole saliva flow (UWS) of more than 0.00 mL/min and chewing-stimulated whole saliva flow (SWS) of more than 0.02 mL/min. Patients with hypertension, diabetes, acute infection of salivary glands, and previous radiotherapy to the head and neck were excluded. All treated patients had signed informed consent documents. This study was approved by the Medical Ethics Committee of Stomatological Hospital Affiliated to Nanjing Medical University (PJ2021-096-001) and registered in the Chinese Clinical Trial Registry (Registration number: ChiCTR2100052314).

### 2.2 Clinical procedure

All patients were recruited by all authors. The randomisation software (www.randomizer.org) randomly assigned participants to the control group, saline group and TA group by a nurse. UWS and SWS, citric acid-parotid Flow (SPF) and Clinical Oral Dryness Score (CODS) were obtained 1 week before the treatment (T0) and 1 week(T1), 8 weeks (T8), 16 weeks (T16) and 24 weeks (T24) after the treatment. All participants were required to complete Xerostomia Inventory (XI) and EULAR SS Patient-Reported Index (ESSPRI) questionnaires on their own at the time mentioned above.

#### 2.2.1 CODS

All included patients were examined for oral dryness by the same physician. CODS was recommended to detect hyposalivation in routine clinical assessment (20). The score observed 10 signs, including mirror sticks to buccal mucosa, mirror sticks to tongue, tongue lobulated/fissured, tongue showing loss of papillae, etc. Those who met the standard of physical signs were scored 1, and those who did not meet the standard were scored 0. The total scores were obtained by summing the scores for all physical signs, and the higher score suggested the more severe xerostomia (21, 22).

#### 2.2.2 XI

All participants were required to independently complete the Xerostomia Inventory (XI) questionnaire concerning xerostomia and oral sensation at the beginning of each outpatient visit. The total score was obtained by summing the scores for the 11 items mentioned above. The score ranged from 11 to 55, and the higher score suggested more severe xerostomia (22–24).

#### 2.2.3 ESSPRI

All participants were required to complete the ESSPRI questionnaire to assess symptoms of pain, fatigue, and dryness on their own at the beginning of each outpatient visit. The changes in two or more points were considered clinically relevant (22, 25).

#### 2.2.4 Sialometry

All patients were subjected to saliva flow detection in a room with the same temperature (21 ± 2°C) and humidity (50%-60%) at the same time in the Department of Oral and Maxillofacial Surgery, Affiliated Stomatological Hospital of Nanjing Medical University. Patients should drink and eat plenty of water before testing and avoid eating, chewing gum, and smoking for 2 hours before testing. The USW measurement was first performed. Saliva was collected from all patients after swallowing. Saliva was spat into the centrifuge tube every 30 seconds and continued to be collected for 5 minutes before weighing. After USW testing, patients were required to chew paraffin slices (Shanghai Dental Materials Factory, Shanghai, China) after swallowing and then spit saliva into a centrifuge tube every 30 seconds. SWS testing was completed after continuous collection for 5 minutes. After that, the SPF was obtained from saliva collection using self-made modified Lashley cups after the lingual drop of citric acid (2% W/V) (26, 27). All saliva samples were measured for salivary volume after centrifugation. Saliva sampling and sample measurement were performed independently by two physicians.

#### 2.2.5 Irrigation

Salivary gland irrigation was performed by the same well-trained oral and maxillofacial surgeon in all participants. During the treatment and follow-up, the specific irrigating drugs used were not disclosed to the patients. Before the treatment, patients should rinse with chlorhexidine gargle for 3 minutes. A dilating probe was used to enlarge the orifice of the parotid and submandibular glands. After the dilation, saline and TA (10mg/mL) were irrigated into bilateral Stensen’s and Wharton’s catheters through orifices of the parotid and submandibular gland using an irrigation syringe. The parotid gland was irrigated with 1.5mL and 1mL for the submandibular gland. Patients should avoid eating and drinking for 2 hours after the irrigation.

### 2.3 Statistical analysis

A sample size of 14 patients per group and primary outcomes were collected referring to previous similar studies (11–13). Data were analysed by using the statistical software SPSS 26.0 for Windows software (SPSS, Inc., Chicago, IL, USA). If the measurement data conformed to the normal distribution and met the homogeneity test of variance, they were represented by Mean ± SD, and two independent sample t-test shall be used to compare the two groups. Two-way repeated measures ANOVA was used for the comparison of outcome indicators. One-way repeated measures ANOVA was used for intra group comparison of time effect. One-way ANOVA was used for inter group comparison at each time point. Bonferroni test was used for *post-hoc* pairwise comparison. Statistical significance was defined as *P*< 0.05.

## 3 Results

**Figure 1:**
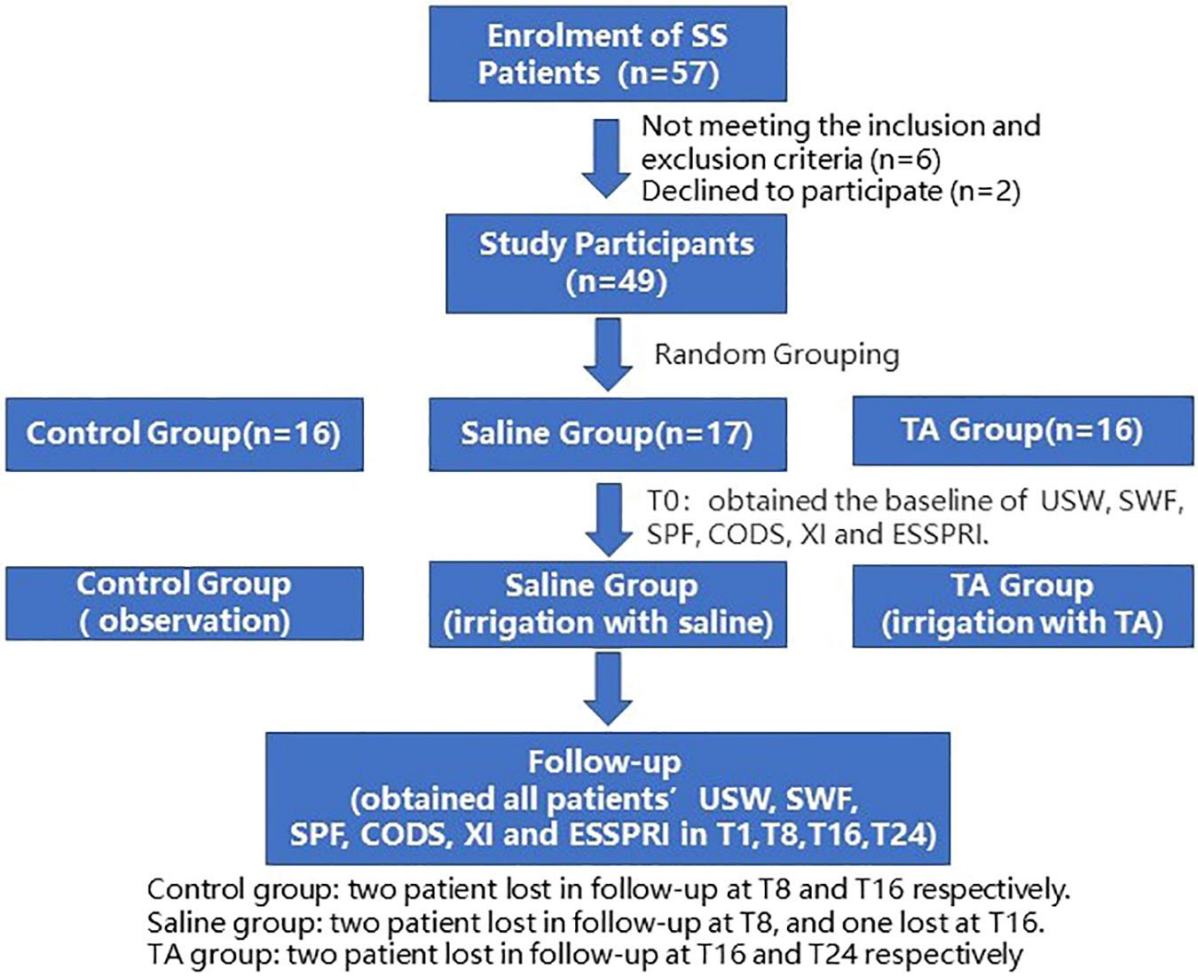
The clinical workflow of this study.

Fifty-seven patients were recruited, and 53 met the inclusion criteria between November 2021 and June 2022. Two of them refused to participate in the study, and seven were lost to follow-up. This trial ended when all patients completed their last follow-up visit (**Figure 1**). Some patients had pain and discomfort in the major salivary glands after irrigation, which were relieved after 72 hours. Twenty-two patients complained of discomfort after irrigation, including 9 cases in the saline group and 13 in the TA group. Thirteen patients complained of pain in major salivary glands after irrigation, including3 cases in the saline group and 10 in the TA group. Among the seven patients who were lost to follow-up, three left their cities of residence, and four had no time for follow-up visits due to personal matters. This study analysed the results of 42patients who had completed follow-up, including 3 males and 39 females, with an average age of 46.07 ± 7.13 years and an average disease duration of 3.48 ± 1.47 years (shown in **Figure 1** and **Table 1**). Other baseline information on the cases is shown in **Table 1**.

**Table 1:** Characteristics of the study population.

Item	Mean (SD) or n (%)
Patients variables	
Age (year)	46.07 (7.13)
Female/male ratio	13:1
Disease duration (year)	3.48 (1.47)
Control group	3.33 (0.89)
Saline group	3.93 (1.83)
TA group	3.14 (1.46)
Primary SS n (%)	28 (66.67%)
Control group	9 (64.29%)
Saline group	10 (71.43%)
TA group	9 (64.29%)
Secondary SS n (%)	14 (33.33%)
Control group	5 (35.71%)
Saline group	4 (28.57)
TA group	5 (35.71%)
Serum antibodies	
Positive anti-SSA	36 (81.82%)
Positive anti-SSB	34 (80.95%)
Positive RF	24 (57.14%)
Positive ANA	20 (47.62%)
Positive labial salivary gland biopsy n (%)	37 (88.10%)
Positive Schirmer test n (%)	36 (85.71%)
Baseline UWS	
Control group	0.14 (0.13)
Saline group	0.14 (0.15)
TA group	0.13 (0.13)
Baseline SWS	
Control group	0.34 (0.43)
Saline group	0.37 (0.40)
TA group	0.37 (0.38)
Baseline SPF	
Control group	0.21 (0.28)
Saline group	0.19 (0.23)
TA group	0.24 (0.34)
XI	42.81 (6.59)
ESSPRI	21.30 (4.18)
ESSPRI (oral dryness domain)	6.57 (1.64)
CODS	4.95 (0.96)
Adverse events after irrigation	
Discomfort	22 (66.67%)
Saline group	9 (52.94%)
TA group	13 (81.25%)
Pain	13 (39.40%)
Saline group	3 (17.65%)
TA group	10 (62.50%)

In the *post-hoc* analysis, all completed follow-up cases in the TA and saline groups were divided into responders and non-responders. The responders refer to patients with T8 SWS/T0 SWS > 1.5. There were 10 responders and 4 non-responders in the TA group respectively. The numbers of responders and non-responders in the saline group were 6 and 8, respectively. Compared with responders, the UWS, SWS, SPF and CODS of T0 in non-responders were significantly increased (*P*<0.05). Compared with responders, the XI and ESSPRI of T0 in non-responders were significantly decreased(*P*<0.05). All the above statistics information is shown in **Table 2**. There were no significant differences in UWS, SWS, SPF, CODS, XI and ESSPRI at all the time points in the control group (*P*>0.05). And all items mentioned above had no significant differences among the three groups at T0 (P>0.05).

**Table 2:** Comparing all outcomes between responders and non-responders at T0.

Item	Responders (*n *= 16)Mean(SD)	Non-responders (*n *= 12)Mean(SD)	*t*-value	*p*-value	Difference (95%CI)
UWS	0.07 ± 0.07	0.21 ± 0.16	-2.945	0.010*	-0.244, -0.040
SWS	0.17 ± 0.21	0.63 ± 0.51	-3.025	0.008*	-0.783, -0.137
SPF	0.11 ± 0.18	0.35 ± 0.34	-2.409	0.028*	-0.468, -0.031
XI	46.19 ± 4.13	38.08 ± 7.24	3.591	0.002*	3.369, 12.852
ESSPRI	23.38 ± 1.59	18.92 ± 4.89	3.151	0.007*	1.423, 7.481
ESSPRI (oral dryness domain)	7.44 ± 1.31	6.08 ± 1.61	2.511	0.018*	0.249, 2.472
CODS	5.19 ± 0.98	4.46 ± 0.88	2.077	0.047*	0.009, 1.443

All p-value<0.05 were marked with*.

### 3.1 Inner-group analysis: Saline group

The UWS of T8 was significantly higher than that at T0 (*P*<0.05). Compared with T0, there was no significant difference in UWS of T1, T16 and T24, respectively (*P*>0.05). The SWS and SPF of T8, T16 and T24 were significantly higher than those of T0, respectively (*P*<0.05). Compared with T0, CODS and ESSPRI of T8, T16 and were significantly decreased respectively (*P*<0.05). However, XI and ESSPRI of T1 were not significantly different from those of T0, respectively (*P*>0.05). Compared with T0, CODS and ESSPRI of T16 significantly decreased (*P*<0.05). However, CODS and ESSPRI of T24 were not significantly different from those of T0 (*P*>0.05). All the above statistics information is shown in **Table 3**.

**Table 3:** Comparison of the indicators among the three groups at different time points.

	Control group			Saline group					TA group						
	Mean(SD)	p-value(vs baseline)	Difference(95% CI)	Mean(SD)	p-value (vs baseline)	Difference(95% CI)	p-value(vs control group	Difference(95% CI)	Mean(SD)	p-value (vs baseline)	Difference(95% CI)	p-value (vs control group	Difference(95% CI)	p-value(vs saline group	Difference(95% CI)
**UWS**															
T0	0.14 (0.13)			0.14 (0.15)			0.888	-0.001(-0.111,0.097)	0.13 (0.13)			0.844	0.01(-0.095,0.116)	0.728	-0.018(-0.120,0.084)
T1	0.11 (0.10)	0.004*	0.028(0.011,0.046)	0.13 (0.12)	0.155	0.017(-0.007,0.042)	0.679	-0.018(-0.108,0.071)	0.11 (0.10)	0.180	0.021 (-0.011,0.052)	0.953	0.003 (-0.088,0.093)	0.629	-0.021(-0.109,0.066)
T8	0.14 (0.11)	0.591	-0.005(-0.023,0.014)	0.19 (0.11)	0.015*	-0.047(-0.083,-0.011)	0.248	-0.049(-0.134,0.036)	0.20(0.10)	0.009*	-0.079(-0.134, -0.023)	0.145	-0.064(-0.150,0.023)	0.732	0.014(-0.069,0.098)
T16	0.13 (0.10)	0.773	0.004(-0.025,0.032)	0.16 (0.14)	0.383	-0.013(-0.045,0.018)	0.609	-0.024(-0.120,0.071)	0.24(0.13)	<0.001*	-0.118(-0.147, -0.089)	0.026*	-0.111(-0.209, -0.014)	0.069	0.087(-0.007,0.181)
T24	0.13 (0.11)	0.406	0.009(-0.014,0.033)	0.14 (0.15)	1.000	<0.001(<0.001,<0.001)	0.725	-0.017(-0.111,0.078)	0.16(0.10)	0.059	-0.031(-0.064,0.001)	0.526	-0.030(-0.126,0.066)	0.765	0.014(-0.079,0.106)
**SWS**															
T0	0.34(0.43)			0.37(0.40)			0.873	-0.027(-0.365,0.311)	0.37(0.38)			0.859	-0.030(-0.374, 0.313)	0.982	0.004(-0.328,0.335)
T1	0.36(0.46)	0.197	-0.021(-0.054,0.012)	0.38(0.44)	0.786	-0.011(-0.093,0.072)	0.921	-0.017(-0.354,0.320)	0.32(0.41)	0.029*	0.054(0.007,0.102)	0.794	0.045(-0.298,0.387)	0.709	-0.061(-0.392,0.269)
T8	0.38(0.44)	0.125	-0.038(-0.089,0.012)	0.47(0.40)	0.033*	-0.102(0.194,0.010)	0.589	-0.090(-0.423,0.245)	0.60(0.47)	0.001*	-0.221(-0.334, -0.109)	0.213	-0.213(-0.555,0.128)	0.454	0.123(-0.206,0.452)
T16	0.35(0.17)	0.899	-0.011(-0.191,0.170)	0.51(0.40)	0.006*	-0.136(-0.227, -0.045)	0.307	-0.152(-0.449,0.145)	0.79(0.50)	<0.001*	-0.416(-0.566, -0.265)	0.006*	-0.435(-0.738, -0.133)	0.056	0.283(-0.008,0.575)
T24	0.33(0.12)	0.891	0.014(-0.201,0.229)	0.52(0.40)	<0.001*	-0.1499(-0.215, -0.083)	0.166	-0.189(-0.460,0.082)	0.67(0.44)	0.001*	-0.296(-0.440, -0.153)	0.017*	-0.341(-0.616, -0.065)	0.256	0.151(-0.115,0.417)
**SPF**															
T0	0.21(0.28)			0.19(0.23)			0.897	0.014(-0.206,0.235)	0.24(0.34)			0.737	-0.037(-0.262,0.187)	0.632	0.052(-0.165,0.268)
T1	0.23(0.31(	0.087	-0.025(-0.055,0.004)	0.23(0.30)	0.245	-0.041(-0.114,0.032)	0.987	-0.002(-0.231,0.227)	0.20(0.29)	0.052	0.041(-0.001,0.083)	0.800	0.029(-0.203,0.262)	0.780	-.031(-0.256,0.193)
T8	0.26(0.32)	0.055	-0.051(-0.103,0.001)	0.28(0.24)	0.003*	-0.087(-0.137,-0.036)	0.837	-0.022(-0.234,0.190)	0.33(0.26)	0.029*	-0.091(-0.171,-0.011)	0.472	-0.077(-0.293,0.138)	0.591	0.056(-0.152,0.264)
T16	0.21(0.12)	0.938	-0.005(-0.132,0.122)	0.28(0.24)	0.005*	-0.090(-0.149,0.031)	0.452	-0.071(-0.261,0.119)	0.51(0.33)	<0.001*	-0.268(-0.365,-0.171)	0.003*	-0.301(-0.494,-0.108)	0.017*	0.229(0.043,0.416)
T24	0.19(0.09)	0.736	0.021(-0.110,0.152)	0.28(0.19)	0.025*	-0.089(-0.165,-0.013)	0.256	-0.096(-0.264,0.072)	0.45(0.31)	<0.001*	-0.209(-0.298,-0.121)	0.003*	-0.267(-0.439,-0.096)	0.042*	0.172(0.006,0.337)
**XI**															
T0	43.38(5.9)			42.00(7.12)			0.589	1.385(-3.763,6.532)	43.14(7.00)			0.926	0.242(-4.990,5.474)	0.650	1.143(-3.905,6.191)
T1	43.69(5.28)	0.487	-0.308(-1.243,0.628)	41.93(6.60)	0.865	0.067(-0.757,0.890)	0.459	1.759(-3.002,6.520)	43.93(6.57)	0.006*	-0.786(-1.301,-0.270)	0.922	-0.2369-5.075,4.603)	0.393	1.995(-2.674,6.664)
T8	42.92(5.95)	0.235	0.462(-0.342,1.265)	39.80(7.36)	0.016*	2.200(0.484,3.916)	0.236	3.123(-2.131,8.377)	38.64(7.07)	0.004*	4.500(1.719,7.281)	0.113	4.280(-1.060,9.621)	0.652	-1.157(-6.310,3.995)
T16	42.69(5.64)	0.133	0.692(-0.243,1.628)	39.80(5.83)	0.034*	2.200(0.190,4.210)	0.213	2.892(-1.726,7.511)	36.93(6.55)	<0.001*	6.214(3.591,8.838)	0.017*	5.764(1.070,10.458)	0.207	-2.871(-7.401,1.658)
T24	42.69(6.06)	0.121	0.692(-0.210,1.595)	39.87(6.37)	0.026*	2.133(0.298,3.968)	0.236	2.826(-1.920,7.572)	38.29(6.12)	0.001*	4.857(2.420,7.295)	0.072	4.407(-0.418,9.231)	0.496	-1.581(-6.235,3.073)
**CODS**															
T0	5.15(0.90)			4.93(1.03)			0.554	0.221(-0.526,0.967)	4.79(0.97)			0.332	0.368(-0.391,1.127)	0.686	-0.148(-0.880)
T1	5.31(0.95)	0.165	-0.154(-0.381,0.073)	4.93(0.96)	1.000	<0.001(-0.296,0.296)	0.289	0.374(-0.330,1.079)	4.64(0.84)	0.336	0.143(-0.166,0.451)	0.068	0.665(-0.509,1.381)	0.400	-0.290(-0.981,0.400)
T8	5.08(0.64)	0.721	0.077(-0.382,0.536)	4.60(0.74)	0.055	0.333(-0.008,0.675)	0.067	0.477(-0.035,0.989)	4.29(0.61)	0.013*	0.500(0.124,0.876)	0.004*	0.791(0.271,1.311)	0.213	-0.314(-0.816,0.188)
T16	4.77(1.09)	0.054	0.385(-0.008,0.778)	4.20(1.52)	0.006*	0.733(0.244,1.223)	0.227	0.569(-0.369,1.507)	4.14(0.95)	0.007*	0.643(0.213,1.073)	0.192	0.626(-0.327,1.580)	0.901	-0.057(-0.977,0.863)
T24	4.92(0.49)	0.337	0.231(-0.272,0.734)	4.66(0.72)	0.104	0.267(-0.062,0.595)	0.358	0.256(-0.301,0.814)	4.21(0.89)	0.006*	0.571(0.198,0.945)	0.016*	0.709(0.142,1.275)	0.102	-0.452(-0.999,0.094)
**ESSPRI**															
T0	21.15(4.54)			20.93(4.83)			0.892	0.221(-3.046,3.487)	21.86(3.21)			0.671	-0.703(-4.024,2.627)	0.563	0.924(-2.280,4.127)
T1	21.15(5.30)	1.000	<0.001(-1.655,1.655)	20.60(5.57)	0.605	0.333(-1.018,1.684)	0.780	0.554(-3.436,4.544)	22.14(4.69)	0.640	-0.286(-1.575,1.004)	0.625	-0.989(-5.045,3.067)	0.430	1.543(-2.370,5.456)
T8	42.92(5.95)	0.708	-0.231(-1.540,1.078)	19.27(5.40)	0.081	1.667(-0.236,3.570)	0.271	2.118(-1.719,5.955)	18.64(4.58)	0.001*	3.214(1.547,4.881)	0.163	2.742(-1.158,6.642)	0.739	-0.624(-4.386,3.139)
T16	20.38(3.20)	0.310	0.769(-0.813,2.352)	17.93(6.18)	0.019*	3.000(0.577,5.423)	0.206	2.451(-1.405,6.308)	13.64(5.03)	<0.001*	8.214(6.251,10.178)	0.001*	6.742(2.822,10.661)	0.027*	-4.290(-8.072,-0.509)
T24	20.31(4.37)	0.076	0.846(-0.104,1.797)	19.13(5.88)	0.120	1.800(-0.533,4.133)	0.542	1.174(-2.683,5.032)	17.36(4.60)	<0.001*	4.500(2.609,6.391)	0.136	2.951(-0.970,6.871)	0.348	-1.776(-5.558,2.007)
**ESSPRI (oral dryness domain)**															
T0	6.00(1.68)			6.80(1.61)			0.204	-0.800(-2.052,0.452)	6.86(1.61)			0.181	-0.857(-2.130,0.416)	0.926	0.057(-1.171,1.285)
T1	5.31(0.95)	0.337	-0.077(-0.245,0.091)	6.60(1.80)	0.082	0.200(-0.029,0.429)	0.455	-0.523(-1.924,0.878)	7.14(1.88)	0.104	-0.286(-0.639,0.067)	0.138	-1.066(-2.490,0.378)	0.429	0.543(-0.831,1.916)
T8	6.08(1.26)	0.753	-0.077(-0.598,0.444)	6.20(1.52)	0.007*	0.600(0.192,1.008)	0.821	-0.123(-1.214,0.968)	6.14(1.46)	0.019*	0.714(0.140,1.288)	0.905	-0.066(-1.175,1.043)	0.915	-0.057(-1.127,1.013)
T16	6.08(1.12)	0.776	-0.077(-0.653,0.500)	6.27(1.44)	0.006*	0.533(0.179,0.888)	0.695	-0.190(-1.162,0.782)	5.29(1.20)	<0.001*	1.571(0.903,2.240)	0.113	0.791(-0.197,1.779)	0.044*	-0.981(-1.934,-0.028)
T24	4.92(0.49)	0.776	0.077(-0.500,0.653)	6.20(1.47)	0.003*	0.600(0.250,0.950)	0.555	-0.277(-1.218,0.664)	5.50(1.09)	<0.001*	1.357(0.821,1.893)	0.377	0.423(-0.534,1.380)	0.133	-0.700(-1.623,0.223)

All p-value<0.05 were marked with*.

### 3.2 Inner-group analysis: TA group

The UWS of T8 and T16 were significantly higher than those of T0, respectively (*P*<0.05). Compared with T0, the UWS of T1 and T24 were not significantly different (*P*>0.05). The SWS and SPF of T8, T16 and T24 were significantly higher than those of T0, respectively (*P*<0.05), but the SWS of T1 was significantly lower than that of T0 (*P*>0.05). The XI of T1 was significantly higher than that of T0, and the XI of other time points were significantly lower than that of T0 (*P*<0.05). The CODS, ESSPRI and ESSPRI (oral dryness domain) of T8, T16 and T24 were significantly decreased compared to those of T0 in the TA group, respectively (*P*<0.05), while those of T1 showed no significant difference compared with T0 (*P*>0.05). All the above statistics information is shown in **Table 3**.

### 3.3 Inter-group analysis: Saline group vs control group

Compared with the control group, UWS of T8 in the saline group was significantly increased (*P*<0.05); there was no significant difference in UWS between the two groups at other time points (*P*>0.05). The SWS and SPF of T8, T16 and T24 in the saline group were significantly higher than those in the control group (*P*<0.05). There was no significant difference between the SWS of T1 in the saline group and that in the control group (*P*>0.05). The XI and ESSPRI(oral dryness domain) of T8, T16 and T24 in the saline group were significantly lower than those in the control group, respectively (*P*<0.05). Compared with the control group, the CODS and ESSPRI of T16 in the saline group significantly decreased (*P*<0.05). There were no significant differences in CODS and ESSPRI between the above two groups at other time points (*P*> 0.05). All the above statistics information is shown in **Table 3**.

### 3.4 Inter-group analysis: TA group vs control group

Compared with the control group, the UWS of T16 in the TA group was significantly increased (*P*<0.05). There was no significant difference in UWS between the two groups at other time points (*P*>0.05). The SWS and SPF of T16 and T24 in the TA group were significantly higher than those in the control group (*P*<0.05). However, there was no significant difference in the TA group’s SWS of T1 and T8 compared with the control group (*P*>0.05). The CODS of T8 and T24 in the saline group were significantly decreased compared with those in the control group, respectively (*P*<0.05). There were no significant differences in the CODS of T1 and T16 between the above two groups. ESSPRI of T16 in the saline group was significantly decreased compared with the control group (*P*<0.05). There were no significant differences in ESSPRI of T1, T8 and T24 between the saline and the control groups (*P*>0.05). There was no significant difference in ESSPRI(oral dryness domain) between the two groups at each time point. All the above statistics information is shown in **Table 3**.

### 3.5 Inter-group analysis: TA group vs saline group

Compared with the saline group, the SPF of T16 and T24in the TA group were significantly increased, respectively (*P*<0.05), while there were no significant differences between the two groups in T1 and T8 (*P*>0.05). Compared with the saline group, the ESSPRI and ESSPRI (oral dryness domain) of T16 in the TA group significantly decreased respectively (*P*<0.05), while ESSPRI and ESSPRI of T1, T8 and T24 showed no significant differences (oral dryness domain) between the two groups. All the above statistics information is shown in **Table 3**.

## 4 Discussion

It has been reported that prednisolone and TA irrigation of the salivary gland can relieve xerostomia in patients with SS (12, 13, 28). Irrigation with TA combined with sialendoscopy was a randomised controlled study (12, 13), while the study using prednisolone was a before-after study on the same patients (28). This study selected TA irrigation without sialendoscopy for a randomised controlled trial.

The reasons why TA’s application has been selected for this study are as follows. Firstly, triamcinolone acetonide hydrochloride injection is a suspension of fine particles and can be precipitated on the ductal inner surface. It can avoid the rapid flushing by the saliva flow, thus prolonging the duration of TA action. Secondly, TA is a moderate-effect glucocorticoid with long-lasting anti-inflammatory effects and relatively fewer side effects, which can be used for a long time. It is suggested that glucocorticoids with the above two characteristics should also be used for irrigation to relieve xerostomia in patients with SS.

In previous similar studies, researchers have applied sialendoscopy to assist TA irrigation in patients with SS (12, 13). During the process, the strictures in the main ducts were removed by sialendoscopy. But the detail of ductal stenosis was not described in the articles. Radionuclide scintigraphy confirmed that the salivary glands in SS had delayed 99mTc intake and were emptied, but no apparent dysfunction of salivary excretion was caused by ductal stenosis (29–31). Sialography and MRI have also confirmed that the main ducts in SS patients were dilatated with no stenosis (16, 17). Consistent with the literature reports, the sialography of the patients in our study also did not show obvious main duct stenosis. Therefore, it is believed that sialendoscopy is not necessary for patients with SS in our study. It is reported that the stenosis in salivary ducts can cause obstructive symptoms such as salivary gland swelling after eating, which usually resolve spontaneously within 30 min (32). In our study, the presence or absence of stenosis of the main duct based on the presence or absence of obstruction symptoms after eating can be roughly determined. Moreover, sialography, MR or ultrasound can be used to determine whether there is stenosis in the main duct for some SS patients with food-induced obstructive symptoms that could not be caused by severe hyposalivation (16, 17, 33). Thus sialendoscopy-assisted treatment may be suggested for patients with definite stenosis of the main duct (33). While for SS patients without significant stenosis in the main ducts, the application of sialendoscopy is not recommended owing to the risk of complications caused by sialendoscopy procedures (18).

For the reasons mentioned above, we believe irrigation without sialendoscopy can achieve similar results. This study has demonstrated that both saline and TA irrigation can increase salivary secretions of patients with SS compared with baseline. Whether TA has a stronger and longer salivary secretion effect than saline is uncertain. This may be caused by the obvious differences in baseline between responders and non-responders. In the TA group, SWS and SPF peaked at 16 weeks. These results have indicated that irrigation without sialendoscopy can still effectively improve salivary secretion in SS patients.

The results of *post hoc* analysis have shown that the non-responders were worse at baseline than the responders. The UWS, SWS, SPF and CODS of T0 in non-responders were significantly increased than those in responders, respectively; while the XI and ESSPRI of T0 in non-responders were significantly decreased than those in non-responders, respectively. This has indicated that the xerostomia of non-responders is more severe before treatment, while that of responders is relatively better. These results showed that patients with non-severe xerostomia had better relief after irrigation than patients with severe xerostomia. The possible reason for this result is that the secretions of salivary glands in patients with severe xerostomia are destroyed, and irrigation could not reverse this situation; For patients with non-severe xerostomia, the irrigation is better due to the retention of the secretions in salivary glands. However, this result is not consistent with the results of previous studies, which showed no significant differences in the baseline between responders and non-responders (12, 13).

Many practitioners face difficulty detecting the orifice and the dilated Wharton’s duct (12, 13). No access to Wharton’s duct can always result in failing treatment. The study by Karagozoglu has demonstrated that about half of patients undergoing sialendoscopy could not gain access to Wharton’s duct (12, 13). Based on our experiences in sialendoscopy, sialography, salivary gland irrigation, methylene blue labelling, and modified dilation probe can help improve the detection rate. The specific steps are firstly drying saliva on the mouth floor, applying methylene blue on the sublingual caruncle, and finally squeezing the submandibular gland to promote saliva outflow. Thus saliva outflow can wash away the methylene blue on the orifice. In addition, the tip of the dilation probe to 15° is also bent so that the rod of the dilation probe could avoid the block of the mandibular incisor for successful accession.

## 5 Conclusion

Irrigation of major salivary glands by TA and saline relieve xerostomia in SS patients. Patients with non-severe xerostomia (responders) have better relief after irrigation than patients with severe xerostomia (non-responders).

## Data availability statement

The original contributions presented in the study are included in the article/**Supplementary Material**. Further inquiries can be directed to the corresponding author.

## Ethics statement

The studies involving human participants were reviewed and approved by the Medical Ethics Committee of Stomatological Hospital Affiliated to Nanjing Medical University. The patients/participants provided their written informed consent to participate in this study.

## Author contributions

YZ, ZF, HD and HW contributed to conception and design. XD, SL, JZ and SL contributed to conception. HD, HW, YCZ, SG, YZ contributed to the acquisition, analysis and interpretation. JZ, HS contributed to the acquisition and analysis. HD, HW, ZF. YZ drafted the manuscript. All authors contributed to the article and approved the submitted version.

## Acknowledgments

The authors of this study would like to thank all the study participants.

## Conflict of interest

The authors declare that the research was conducted without any commercial or financial relationships that could be construed as a potential conflict of interest.

## Publisher’s note

All claims expressed in this article are solely those of the authors and do not necessarily represent those of their affiliated organizations, or those of the publisher, the editors and the reviewers. Any product that may be evaluated in this article, or claim that may be made by its manufacturer, is not guaranteed or endorsed by the publisher.
