# Supplementary material for: A randomized controlled trial to verify the irrigation of salivary glands in relieving xerostomia in patients with Sjögren’s syndrome
Source: Front Immunol. 2022 Nov 10;13:1039599. doi: 10.3389/fimmu.2022.1039599 (PMC9684172; doi:10.3389/fimmu.2022.1039599)
Supplement: Supplementary file 1 [file DataSheet_1.doc]

**
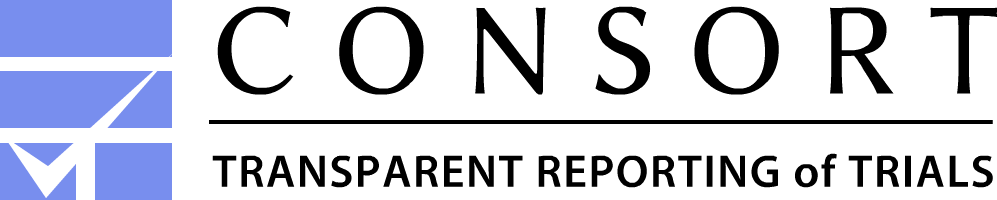
**

**CONSORT 2010 Flow Diagram**

**Enrollment**

**Allocation**

**Follow-Up**

**Analysis**

Assessed for eligibility (n=57)

Excluded (n=6)

♦  Not meeting inclusion criteria (n=4)

♦  Declined to participate (n=2 )

♦  Other reasons (n=0)

Analysed (n=14)
♦ Excluded from analysis (give reasons) (n=0)

Lost to follow-up (give reasons) (n=2)

Discontinued intervention (give reasons) (n=0)

Allocated to control group (n=16)

♦ Received allocated intervention (n=16)

♦ Did not receive allocated intervention (give reasons) (n=0)

Randomized (n=51)

Allocated to saline group (n=17)

♦ Received allocated intervention (n=17)

♦ Did not receive allocated intervention (give reasons) (n=0)

Allocated to TA group (n=16)

♦ Received allocated intervention (n=16)

♦ Did not receive allocated intervention (give reasons) (n=0)

Lost to follow-up (give reasons) (n=2)

Discontinued intervention (give reasons) (n=0)

Lost to follow-up (give reasons) (n=3)

Discontinued intervention (give reasons) (n=0)

Analysed (n=14)
♦ Excluded from analysis (give reasons) (n=0)

Analysed (n=14)
♦ Excluded from analysis (give reasons) (n=0)
